# “Surrounded, detached”: the relationship between defensive peripersonal space and personality

**DOI:** 10.3389/fpsyt.2023.1244364

**Published:** 2023-10-13

**Authors:** Monica Biggio, Andrea Escelsior, Martino Belvederi Murri, Alice Trabucco, Federico Delfante, Beatriz Pereira da Silva, Ambra Bisio, Gianluca Serafini, Marco Bove, Mario Amore

**Affiliations:** ^1^Department of Experimental Medicine, Section of Human Physiology and Centro Polifunzionale di Scienze Motorie, University of Genoa, Genoa, Italy; ^2^IRCCS Ospedale Policlinico San Martino, Genoa, Italy; ^3^Department of Neuroscience, Rehabilitation, Ophthalmology, Genetics, Maternal and Child Health (DINOGMI), Section of Psychiatry, University of Genoa, Genoa, Italy; ^4^Institute of Psychiatry, Department of Neuroscience and Rehabilitation, University of Ferrara, Ferrara, Italy

**Keywords:** PID-5, Hand Blink Reflex, embodied cognition, individual difference factors, network analysis

## Abstract

**Introduction:**

Personality shapes the cognitive, affective, and behavioral interactions between individuals and the environment. Defensive peripersonal space (DPPS) is the projected interface between the body and the world with a protective function for the body. Previous studies suggest that DPPS displays inter-individual variability that is associated with psychiatric symptoms, such as anxiety. However, DPPS may share a link with personality traits.

**Methods:**

Fifty-five healthy participants were assessed with the Personality Inventory for DSM-5 (PID-5)–Adult to evaluate personality dimensions. Subjects underwent the Hand Blink Reflex (HBR) task that estimates the DPPS limits by assessing the modulation of blink intensity in response to the median nerve stimulation. Data of the HBR was analyzed with Bayesian multilevel models, while the relationship between DPPS and personality traits was explored using network analysis.

**Results:**

HBR was best modeled using a piecewise linear regression model, with two distinct slope parameters for electromyographic data. Network analyzes showed a positive correlation between the proximal slope and detachment personality trait, suggesting that individuals with higher scores in the detachment trait had an increased modulation of HBR, resulting in a larger extension of the DPPS.

**Discussion:**

Features of the detachment personality trait include avoidance of interpersonal experiences, restricted affectivity, and suspiciousness, which affect interpersonal functioning. We suggest that DPPS may represent a characteristic feature of maladaptive personality traits, thus constitute a biomarker or a target for rehabilitative interventions.

## 1. Introduction

The human body is virtually surrounded by a peri-personal space (PPS), the portion of space lying in the boundary zone between reachable objects near the body and the body itself (1, 2). The PPS has an essential role distinguishing the self from others (3), and discriminating the location of external stimuli relative to the position of one’s own body. Ultimately, the PPS serves the individual interaction with the surrounding space (3). Defensive peripersonal space (DPPS) has been defined as the part of the PPS where there is increased physiological reactivity to stimuli that are potentially harmful to the body (4, 5). The DPPS can be investigated using the Hand Blink Reflex (HBR) task (6–8) which measures the changes in orbicularis oculi muscles contraction in response to an electrical stimulation of the median nerve when the arm is placed in different positions with respect to the head. In particular, in a static position of the arm, the HBR is modulated by the hand position in space: the response dramatically increases when the stimulated hand is located close to the face, inside the DPPS (8). Further, in dynamic conditions (i.e., when subjects performed upper limb voluntary movements toward and far from the face), the DPPS boundary around the body is continuously shaped by the predictive motor system, resulting in an increased HBR response when the hand moves toward the face, but decreases when the hand moves away from the face (7). This shows that in dynamic conditions HBR modulation depends not only by the actual position of the stimulated hand, but also by the final position where the hand is expected to be at the end of the movement.

HBR is in fact related to the perceived “threat” for the face posed by the electrical stimulation of the wrist, and is therefore modulated by those conditions that modulates the dangerousness of a stimulus. For example, when a wooden screen is placed between the participants’ face and their hand the HBR enhancement by hand-face proximity is suppressed (9), whereas merely limiting the visual component (i.e., keeping the eyes closed) leads to an increase in the intensity of the reflex when the hand is inside the DPPS (8).

Studies of the DPPS in adults show significant interindividual differences, although little is still known about the determinants of such variability. Ronga and colleagues showed that the PPS is already present hours after birth (10), and recently (11) has been demonstrates an association with adult attachment style (attachment anxiety specifically) and the PPS flexibility to adapt situationally. The DPPS may display similar changes across developmental phases. Accordingly, Fossataro and colleagues suggests that early attachment experiences have an impact on how the boundaries of DPPS are encoded, finding a different HBR modulation in a group of participants with organized interpersonal cognitive schemas about the early attachment experiences with respect to a group with disorganized one (12). It has been found that not only early, but also late experiences related to sport activity can modify threat perception, and thus the extension of the DPPS, also measured through HBR recording, which was shaped by expertise in boxing athletes (13). Moreover, studies found that the DPPS displayed inter-individual differences in relation to psychopathology, such as the levels of arousal in phobic subjects (14) and the severity of anxiety (15, 16). Two other studies examined the relationship between specific traits related to interpersonal functioning, namely social cognition (17), and empathy (18). To our knowledge, however, the DPPS has never been investigated in relation to personality. Personality is defined as the “*pattern of behaviors, feelings, and thoughts, that shapes specific forms of interactions between the subject and its environment, determined both by genetic and neurobiological systems and by the experiences*” (19). Modern accounts of personality conceptualize it as comprising multiple dimensions spanning affective, cognitive, and behavioral domains, shaped by the interaction of innate predisposition and experience. In this framework, sensorimotor, embodied processes related to the self and spatial navigation may be essential components that influence, or are influenced by, the development of personality, especially in interpersonal and social functioning (20).

Given these premises, the aim of this study was to examine the relationship between the variability of the DPPS extension, measured by the HBR response, and personality facets in human subjects. We did not have predefined hypotheses regarding the association between specific personality traits and DPPS, hence we used and explorative approach. We expected, in fact, that parameters of the behavioral task and self-report measures to be highly inter-correlated within each domain, but weakly correlated cross-domain (21). Thus, we used network analyzes, an exploratory multivariate approach, to examine the association between parameters of the peripersonal space and personality scores from the PID-5. Thus, our aim was to investigate the relationship between the DPPS and personality in humans through a network analysis. In particular, we explored whether the extension of the DPPS, measured as the increase of HBR response when the hand is placed near the face, was associated with specific personality traits.

## 2. Materials and methods

### 2.1. Participants

We recruited 55 participants (37 females and 18 males, mean age: 29.3 ± 7.19 years) between February and September 2021.

Inclusion criteria were: age higher than 18 years; fluent in Italian; willingness to participate in the study.

Exclusion criteria were: history of psychiatric disorders; first-degree familiarity for psychiatric disorders; neurological and major medical illnesses; history of substance use disorder; history of alcohol abuse; obesity and orthopedic problems for the right-dominant hand. Handedness was determined with the Edinburgh Handedness Inventory (22). Demographic characteristics of the sample are reported in Table 1. In the sample, we gathered information related to age, sex, marital status, employment, educational level, and BMI. We collected information on BMI because it can influence nerve conduction and sensory perception. Specifically, obesity or its comorbidities have been shown to affect median nerve conduction (23, 24), and the distribution of body fat can impact pain perception (25).

**Table 1:** Demographic characteristics of the sample.

Characteristics	Total participants (*N* = 55)
Age, mean (SD), y	29.30 (7.19)
Female sex, *N* (%)	37 (65.5)
Married, *N* (%)	5 (9.1)
Working, *N* (%)	28 (50.9)
Educational Level, mean (SD), y	17.30 (2.11)

The study was approved by the local ethics committee (Comitato Etico Regionale - CER Liguria: 633/2020 - DB id 11,064) and conducted in accordance with the Helsinki Declaration, and carried out in agreement with legal requirements and international norms (26). All subjects gave informed consent for participation in the study after receiving a comprehensive explanation of study procedures and goals.

### 2.2. Experimental setup and procedure

The HBR response was elicited by administering transcutaneous electrical stimuli to the median nerve at the right wrist, using a surface bipolar electrode attached with a velcro strap and connected to a Digitimer constant current stimulator (DS7AH HV, Digitimer Ltd., United Kingdom). Stimulus intensity was adjusted to elicit in each participant clear HBR responses (mean stimulus intensities were 66.24 ± 25.21 mA, range 22–99.9 mA). In 37 subjects the post–stimulus activity was more than two standard deviation higher than the post – stimulus activity, hence they were considered responders (*circa* 67% of the population) (27). Participants did not report painful sensations elicited by the stimulation. The duration of the stimulus was 200 μs and the inter-stimulus interval was ~30 s. A twin-axis electronic goniometer (TSD130B, BIOPAC System, Inc.) connected to a BIOPAC MP100 system was used to measure and record the elbow angle of the three target stimulation positions. EMG activity was recorded by means of two MP100 BIOPAC EMG channels from the orbicularis oculi muscles bilaterally, using two pairs of bipolar surface electrodes with the active electrode over the mid lower eyelid and the reference electrode laterally to the outer canthus. Signals were amplified and digitized at 1 kHz (BIOPAC MP100).

Participants were seated in front of a table in order to keep the right elbow leaning at the limit of it, in a position allowing the stimulated wrist to be in front of the ipsilateral eye. The electrical stimulation was delivered while the participant’s stimulated hand was located at one of three target positions: (i) when the elbow angle was 10° less than the maximal arm extension (FAR) at about 40 cm from the face, (ii) the half of the difference between the angles of maximal arm extension and flexion (MIDDLE) at about 20 cm from the face, (iii) and when the angle was 10° more than the maximal elbow flexion (NEAR) at about 4 cm from the face (7, 28). Participants were instructed to keep their gaze on a fixation point placed at 60 cm from the eyes throughout the experiment.

**Figure 1:**
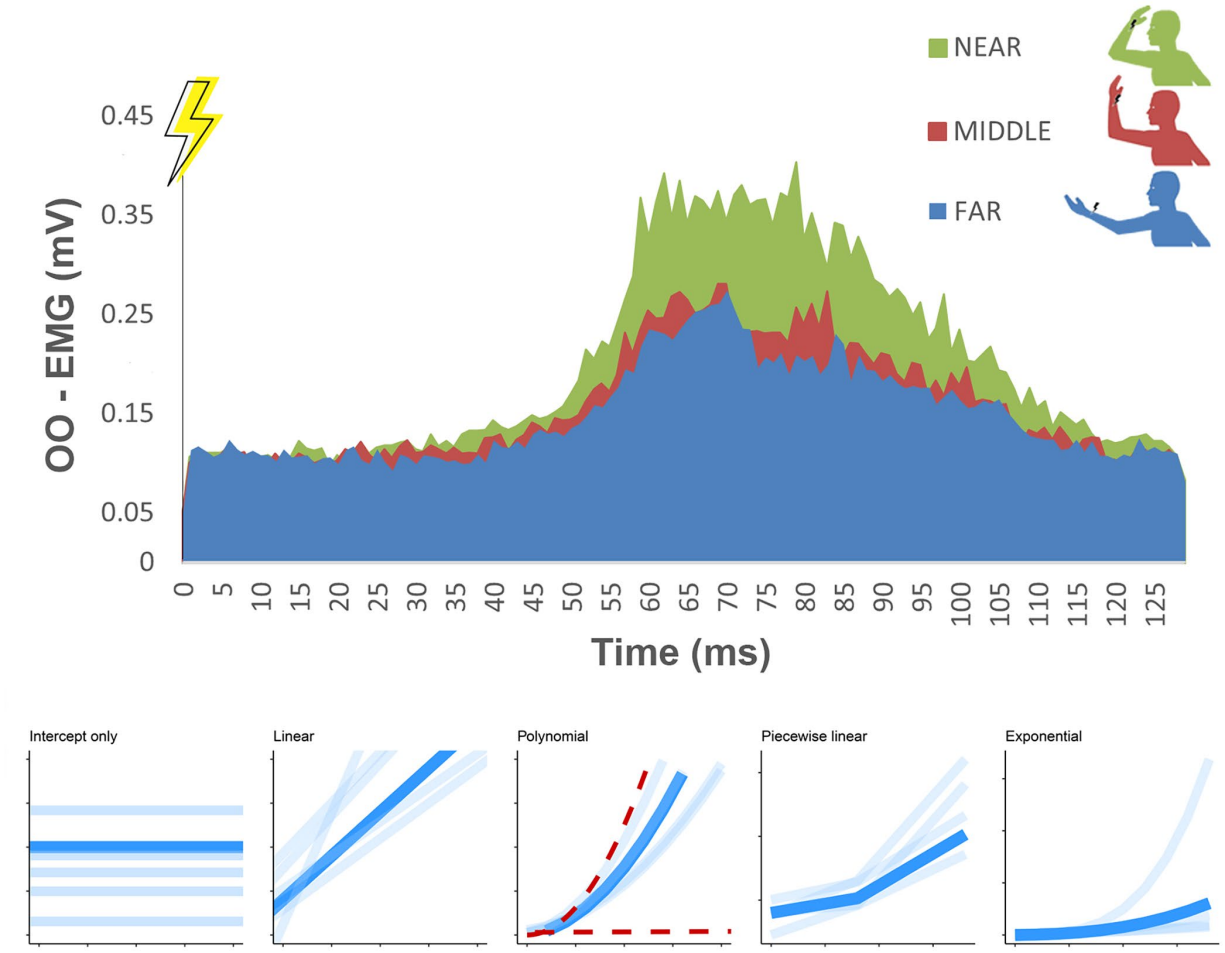
Group-average, rectified HBR waveforms recorded from Orbicularis Oculi muscles (OO) and averaged for left and right eyes when the arm was placed in the three stimulation positions: FAR (blue), MIDDLE (red) and NEAR (green). The target positions are illustrated in the upper right corner, showing the location of the static arm when the electrical stimulations were provided (corresponding to time = 0). Area under curves (AUC–mV*ms) were computed for each position. In the lower panel are represented different HBR estimated models of fit from the Bayesian analysis starting from a “null” intercept-only model to a more complex exponential model with three model estimates parameters: intercept, multiplicative and exponential parameter.

Participants were instructed, trial by trial, to put the arm in one of the three positions previously identified (as shown in Figure 1). The order of the hand positions at which the participant received the electrical stimulus was pseudo-random. The subject received then the electrical stimulation after a randomly variable delay, 0 to 3 s after the subject had stably assumed the target position. Fifteen acquisitions were collected for each side of stimulation, 5 for each hand position. In the case of no blink response from the subject, the trial was still averaged between the other trials. If, on the other hand, the EMG trace should have been fouled by artifacts or contractions of the subject’s face (e.g., a yawn), the order of the trials was continued and the problematic trial was repeated at the end.

### 2.3. Data processing and model estimation

EMG signals were processed with a custom-made MatLab software (MathWorks, MA, United States), and HBR signals from each participant were filtered and rectified. Participants’ responses were averaged separately in each condition and for each participant. Trials with an abnormal EMG activity preceding the HBR response were discarded during the analysis, for a total of less than 1% of the total trials. The area under the curve (AUC, mV*ms) of each HBR average waveform was considered as the HBR outcome parameter. To compute the AUC in each average EMG trace the software automatically analyzes a 130 ms-time interval from the stimulus onset that always contained the participant’s blink (see Figure 1). The resulting curve was then integrated to compute the AUC. In all experiments, data were averaged across ipsilateral and contralateral recording sides (right and left eyes).

To determine the best model to estimate HBR responses, we analyzed our data using a Bayesian multilevel approach, which accounts for inter-individual variability and retains full information on parameter uncertainty. Bayesian analyzes generally produce stable and accurate parameter estimates that may reflect the data generating process (29). The dependent variable was AUC data measured at the three angles of stimulation, nested within subjects. We fitted models of increasing complexity, starting from (1) a “null” intercept-only model (Figure 1, lower panel). This model recovers one parameter for each subject, assuming the HBR AUC does not vary across different positions of the arm. Then, we fitted (2) a linear model that assumes that the AUC value varies with a constant linear trend across arm positions. This model estimates two parameters: intercept and slope. The former can be interpreted as the baseline AUC level measured at the most distant position of the arm, the latter the steepness of increase (or decrease) of the AUC. We then tested (3) a quadratic polynomial model, assuming the AUC results from the sum of linear and quadratic changes across positions (three parameters: intercept, linear slope and quadratic slope parameters), and (4) a piecewise linear model. This assumes the AUC increases according to two distinct linear slopes (three parameters: intercept, FAR-to-MIDDLE position (FtoM) slope, MIDDLE-to-NEAR position (MtoN) slope). *A priori*, the piecewise linear model was hypothesized to be parsimonious and consistent with the experiment characteristics, given previous studies on peripersonal space (28). Compared to the linear model, the piecewise model is less restrictive since it allows different rates of change of the AUC related to the proximity of the hand to the face (i.e., between the FtoM and the MtoN), which corresponds to the typical HBR response. In the piecewise model, the intercept parameter can be interpreted as the baseline AUC, measured in the farthest position, the first slopes parameter can be interpreted as the rate of change of the HBR response between the far and middle position, and the second slope parameter as the rate of change between the middle and the near-face position. Lastly, we fitted (5) an exponential model (three parameters: intercept, multiplicative and exponential parameter). We used a lognormal family distribution for the intercept, linear, polynomial, and piecewise models. For the exponential model, we used a normally distributed response. Bayesian multilevel analyzes were fit using the 2.16.1 *brms* package, which is a front-end for the Stan program (29). Models were run each with 4 chains, each with 1,000 warmup iterations and 5,000 iterations. Models were compared using Leave-One-Out (LOO) cross-validation, which is indicative of out-of-sample prediction accuracy. We also report Bayesian R^2^ values. Median parameter values of each participant from the best fitting model would be used for further analyzes.

### 2.4. Assessments

Each participant completed the Personality Inventory for DSM-5 (PID-5) (30), full version for adult, a questionnaire, developed to assess the Criteria B (Pathological Personality Traits) in section III of the DSM-5 with the aim of adopting a dimensional and inferential-contextual model, the Alternative Model for Personality Disorders (AMPD). The AMPD offering an approach beyond traditional personality disorder categorization, focusing on objective trait assessment essential for precise diagnoses (31, 32). The AMPD provides a systematic way to identify and measure personality psychopathology, crucial for neurobiological studies on maladaptive personality differences (33, 34). The original version of the PID-5 showed good evidence of validity and reliability. It has 220 items with a Likert scale from 0 (Very False or Often False) to 3 (Very True or Often True). It assesses 25 personality facets, sub-dimensions that can be combined to form 5 main trait domains: negative affect (NA), detachment (DET), antagonism (ANT), disinhibition (DIS), and psychoticism (PSY). These domains can be considered as the polar opposites of normal personality dimensions, according to the most authoritative theoretical models of personality, such as the Five Factor Model (35) and The Personality Psychopathology-5 (36).

### 2.5. Network analysis

**Figure 2:**
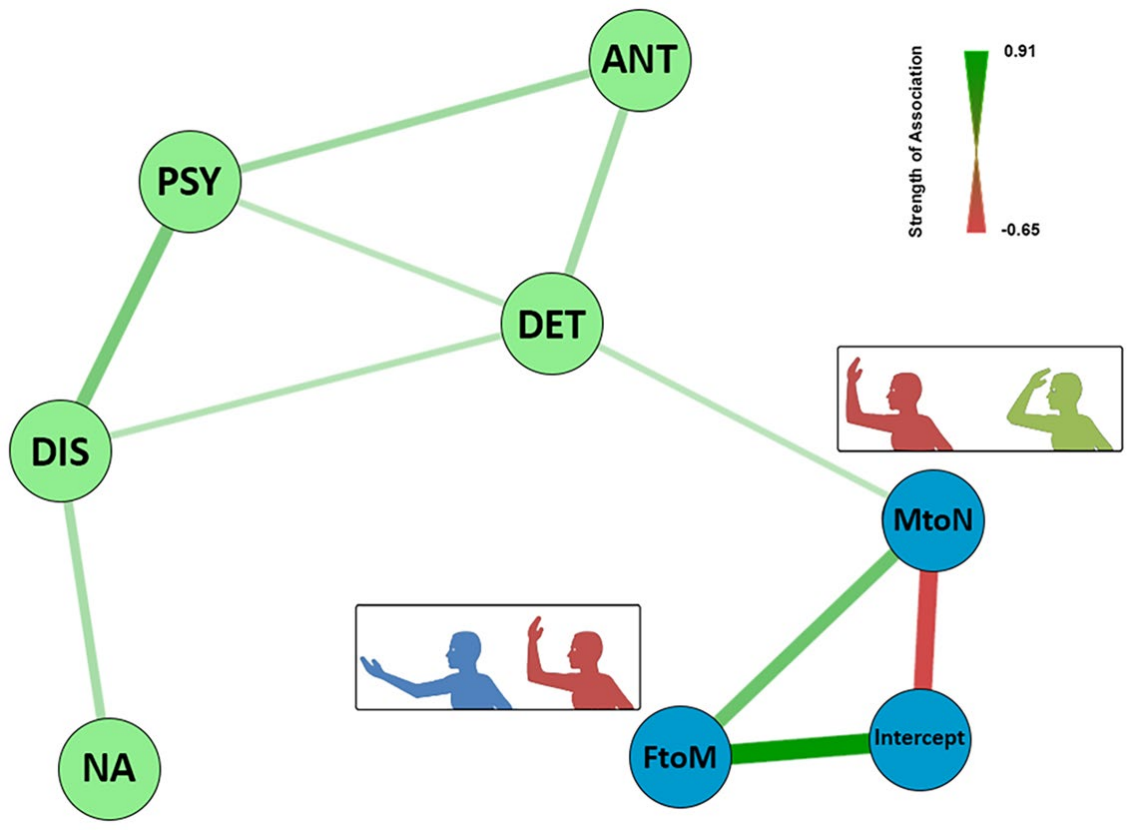
Network of the associations between personality traits and DPPS extension. The thickness of edges reflects the magnitude of the correlation. Green lines represent positive correlations, red lines represent negative ones–legend of the strength of association is reported on the top-right of the figure. Green nodes represent personality measures, derived from PID–5, blue nodes represent parameters derived from the piecewise linear model of HBR data. NA: indicates Negative Affectivity; DIS indicates Disinhibition; DET indicates Detachment; ANT indicates Antagonism; FtoM indicates the slope calculated between HBR response obtained in far and middle position; MtoN indicates slope calculated between HBR response obtained in middle and near; intercept indicates values assumed by the variable when FtoM and MtoN values have mean of 0.

To explore the association between personality trait(s) and measures of peripersonal space, we perform a network analysis. We expected and high inter-correlation within HBR domain and personality domain, but we did not have predefined hypotheses regarding the cross-domain association. Thus, we used network analyzes, an exploratory multivariate approach, to examine the association between parameters of the peripersonal space and personality scores from the PID-5. Network analyzes have been successfully used to model multiple causal dependencies between psychopathology and other data (37, 38). In particular, the Gaussian Graphical Model (GGM) estimates conditional dependencies (similar to partial correlations) between multiple variables of interest, after adjusting for all variables in the model. In a network, variables are visualized as nodes and the strength of their unique association is represented as a green (positive) or red (negative) edge of varying thickness (Figure 2). The GGM was estimated using the non-regularized Bayesian approach implemented in the R package BGGM 2.0. This method is more conservative than using regularized network analysis and is based on a Wishart prior distribution for the correlation matrix. Posterior samples were used to estimate credibility intervals on each correlation, to test the probability that each parameter is non-zero. We used a slightly broader threshold than conventional values (85% instead of 95%) considering the relatively small sample size and the expectation of low association magnitude.

## 3. Results

### 3.1. Sample description

Table 2 reported the 55 participants mean scores for each PID-5 dimension.

**Table 2:** PID-5 dimensions scores.

PID-5 Domains	Mean (SD) score
Negative affectivity	0.98 ± 0.56
Detachment	0.60 ± 0.46
Antagonism	0.57 ± 0.46
Disinhibition	0.66 ± 0.50
Psychoticism	0.47 ± 0.39

To explore the association between HBR and personality dimension, the mean scores of all the participants for each domain from the PID-5 have been entered into the network.

### 3.2. HBR data modeling

First, we verified that the physiological characteristics of HBR were respected in terms of area under the curve, duration and latency. The results of the analysis are reported in the Supplementary materials and are in line with the literature (7, 8). All multilevel models converged well. High inter-individual variability was captured by wide credible intervals for group level standard deviations. The piecewise linear model had the best out-of-sample prediction performance: the lowest Leave-One-Out cross-validation Information Criterion (LOOIC) and the highest Expected Log Predictive Density (ELPD) values. All models explained high proportion of variance of HBR data (Bayesian *R*^2^ Intercept only: 74.7%; Linear: 86.7%; Polynomial: 88.7%; Piecewise linear: 89.1%; Exponential: 92.4%). The piecewise linear model parameters are displayed in Table 3. There was greater uncertainty for the first slope parameter (the 95% interval crossed 0) than for the second slope parameter (95% probability of being positive). Posterior predictive checks indicated that the model response distribution was appropriate and fit data well.

**Table 3:** Parameters of the Piecewise linear model of HBR data.

Parameter	Estimate	SE	l-95% CI	u-95% CI	Rhat	Bulk_ESS	Tail_ESS
Intercept	2.72	0.07	2.59	2.86	1	1,746.88	3,666
FtoM	0.02	0.03	−0.05	0.09	1	9,931.17	10,063
MtoN	0.18	0.06	0.07	0.29	1	12,172.54	11,027
sigma	0.16	0.02	0.12	0.19	1.01	866.46	469

### 3.3. Network analysis

As expected, network analysis recovered a structure where the constructs of maladaptive personality domains (Negative Affectivity, Detachment, Antagonism, Disinhibition, Psychoticism) and HBR parameters (Slope FtoM, Slope MtoN, corresponding to the steepness of change of HBR intensity related to the proximity of the hand to the face, and intercept) formed two distinct patterns with strong intrinsic connections. The two domains were connected through an edge between the nodes *Detachment* and *MtoN slope*. In particular, the Negative Affectivity is only connected with the Disinhibition domain, which is, strongly connected with Psychoticism and mildly with Detachment.

Antagonism, Psychoticism and Detachment are, themselves, interconnected. The HBR cluster showed a direct relation between the two slopes indicating the relation with the two different DPPS portions. Also, intercept value correlates positively with the space external to DPPS, namely FtoM, and negatively with the inside portion of DPPS, namely MtoN. This may suggest that HBR at baseline influence the growth of the response in the DPPS boundary. Indeed, the higher the farther AUC, the higher the steepness of FtoM, leading a saturation (flat slope) in MtoN (extended DPPS) On the contrary low basal HBR levels show reduced growth in middle, but a remarkable increase in MtoN slope (restricted DPPS).

## 4. Discussion

The aim of the study was to explore the relationship between personality facets and DPPS. Using the slope of HBR response intensity as a measure of DPPS extension, we considered two portions of space, one directly surrounding the face and one toward their hand. We found a positive association between MtoN slope, namely the extension of the DPPS near the subject face and the Detachment personality dimension, so that a steeper boundary of the DPPS was related to personality with a higher propensity to detachment. Instead, personality was not connected with the FtoM slope or the intercept parameter, suggesting that it may bear less or no association with basal EMG activity or with the HBR evoked in the far portion of space.

The personality dimension of Detachment corresponds to a stable behavioral pattern with a tendency to avoid emotional experiences. Detachment includes both withdrawing from interpersonal interactions ranging from casual, daily interactions to close friendships or intimate relationships, as well as a restricted experience and expression of affect (39). The domain of Detachment derives from the facets of withdrawal, intimacy avoidance, anhedonia, depressivity, restricted affectivity, and suspiciousness (39). Detachment is intended as the need for greater interpersonal distance and tendency to avoid social contacts (40, 41). Developmental accounts of individuals with detached personality have been associated with a poor sense of security to self and others and insecure attachment styles, possibly related to poor parental bonding in the early phases of life (42). Interestingly, no correlation was found between Detachment and a steeper FtoM slope, thus suggesting a relationship with broader DPPS boundaries rather than a gradual increase of HBR. Our findings suggest that people with more pronounced Detachment attitudes display an over-responsive attitude toward external threatening stimuli, as indicated by a steeper proximal DPPS. Thus, the Detachment personality trait may constitute a learned compensatory mechanism gradually implemented since the early experiences to adult life to minimize the risk of contact with external stimuli with potential threatening valence. Knowledge on the relationship between the PPS and psychopathology is still limited and often contradictory, especially considering the more specific DPPS. To our knowledge, our study is the first to employ a comprehensive, reliable measure of personality, a construct that includes several inter-related features. Our finding is consistent with, and extend the findings of previous studies on specific psychological or psychopathological traits, showing that healthy individuals with high empathy trait (18), disorganized attachment styles (12) high anxiety trait (43, 44) or claustrophobia (43, 45) can modulate the PPS extension. Intriguingly, both a transitory state like anxiety and a chronic condition like claustrophobia were linked to an enlarged PPS size, with reduced plasticity associated to the phobic disorder (28, 43, 45).

The question here is whether certain personality domains can affect the representation of one’s DPPS or if how specific characteristics of the PPS underlie the etiology of maladaptive personality traits (such as Detachment). A first hypothesis is that maladaptive personality traits, intended as pervasive interior modality of thinking, behaving, and relating with others, can lead to a reduced extension of the DPPS, which becomes less reactive to external influences or conditioned by a dysfunctional interpretation of them. As a larger DPPS size is strictly associated with the necessity to avoid interpersonal contact and on defensive purpose, its positive correlation with the detachment dimension results coherent with the underpinned personality profiles and their defensive mechanisms.

On the other hand, PPS directly contributes to the experience of self and self-boundaries, through tactile processing and multisensory interactions, encoding the experience of body integrity (46), and of the body ownership (body self-consciousness) (47), corroborating the stability (or instability) of personal identity over time. We may hypothesize that early experiences could shape DPPS extension, that could become stable associated with hypervigilance and attentional bias characteristics. The Detachment, intended as the need of greater interpersonal distance (40, 41), would be a defensive compensation for the feeling of invasion of one’s intimacy. Interestingly, von Mohr and colleagues (11) explored the extension on PPS and interpersonal space (48) in subject with attachment anxiety, which itself is characterized by negative biases in the interpretation of social cues and worry about emotional closeness (49). In particular they found that in subjects reporting high levels of attachment anxiety, PPS boundaries are always very sharp, whereas in subjects with low scores, PPS boundaries are modulated situationally: sharper in the presence of strangers and less defined when subjects are alone. Authors suggests that the “hypervigilant strategy” adopted by individuals more anxiously attached may be an adaptive response developed in response to an inadequate childhood environment and then consolidated over time. Although this study focused on a more social aspect, the results seem in line with our association between detachment and more defined DPPS boundaries.

While the Detachment is a stable personality trait, a crucial characteristic of the PPS is represented by its plasticity and dynamicity: plasticity refers to the flexibility elicited through training or learning, while dynamic changes are elicited in response to modifications in the environment or to the internal state changes of an individual, including emotional state changes (46). Therefore, PPS is continuously remapped by both top-down and bottom-up interoceptive and exteroceptive stimuli (50). As a safety boundary around the body, DPPS is dynamically shaped through predictive motor mechanisms (7), with an increased HBR when the stimulus is near to the face (7, 8). Given this premise, it can be assumed that the body-space interactions vary depending on individual’s purpose (reaching or defensive), despite an identical spatial encoding of the incoming stimulus (51). Therefore, DPPS is modulated by several factors: interpersonal interaction and levels of empathy can affect subcortical defensive responses as measured by the HBR (18).

A seminal work of Sambo & Iannetti (28) found a correlation between a larger DPPS extension and higher trait anxiety, measured with STAI (Sate Trait Anxiety Inventory) (52), classifying subjects depending on the size of their DPPS with fixed hand-face distances (e.g., “ultra-far,” 60 cm; “far,” 40 cm; “near,” 20 cm; “ultra-near,” 4 cm.). On the contrary, we did not find any significant correlation between DPPS extension and the PID-5 negative affect dimension, which comprises the anxiety facet. Inconsistency between these findings may depend on classification of subjects and on assessment instruments. We choose to consider a continuous indicator of DPPS extension, i.e., the two FtoM and MtoN slopes, to continuously order the responses of our subjects. On the other hand, Sambo and colleagues tried to define 4 limited classes of behavior based on HBR responses. Further, the personality assessment chosen in the two works are very different. In particular, the STAI anxiety trait encompasses different anxiety symptoms, including anticipatory, somatic, affective and cognitive symptoms (52). On the contrary, the anxiety facet of the PID-5 focuses on anticipatory anxiety (e.g., “I worry a lot about terrible things that might happen,” “I get very nervous when I think about the future,” “I always expect the worst to happen”) (53). Future studies might clarify whether enlargement of the DPPS extension is specifically associated with a specific tendency to experience somatic symptoms of anxiety, in particular hyperarousal, which has been associated with an increased functioning of the human defensive system (54). In fact, the personality domain of Detachment has been associated with avoidant, obsessive-compulsive, and schizotypal personality disorders (55) and with PTSD, particularly the internalizing subtype (56). These disorders share a tendency to develop symptoms of hyperarousal in response to various perceived threats.

This study is strengthened by a relatively large sample size, as regard as HBR topic, a rigorous modeling of DPPS data and a reliable assessment of personality. However, we cannot draw inferences on the causal association between DPPS extension and psychopatological traits, due to the cross-sectional design. While personality domains are hypothesized to be stable in the long-term, the PPS may be plastic, thus possibly reflecting training or learning from adaptively responding to the environment (46). In this framework, the PPS three-dimensional space modulable by environmental or organic stimuli (50). Further longitudinal studies are needed to disentangle the relationship between DPPS and personality. If the DPPS was confirmed as a determinant of maladaptive personality traits, it may constitute a biomarker or a target for rehabilitative interventions. This work presents, however, some limitations. While this can be considered a rather large study in the specific HBR literature, the sample size may have been insufficient to capture small effects with sufficient statistical power. Further studies are still required to estimate what size of effects would correspond to significance in this field. Even if this is, to our knowledge, one of the largest studies regarding the analysis of HBR data, the sample size is relatively small for the investigation of the personality dimensions and is represented by a non-clinical population. Another critical point may regard the questionnaire chosen for the personality assessment. Despite the PID-5 being a validated tool for scientific research and the assessment of personality traits, dimensions, and disorders, its relatively recent introduction may potentially limit the reliability of results.

This study also presents an exploratory approach, since we did not have *a priori* hypotheses, and further investigations may be necessary to investigate more precisely the correlation between the detachment, its features and DPPS.

In conclusion, we suggest that inter-individual differences in DPPS extension may be partially explained by personality trait. This suggest that maladaptive aspect of personality can affect the experience we have of the world, conditioning how we learn to which stimuli react, thus modulating the expression of our “safety margin” (57).

## Data availability statement

The raw data supporting the conclusions of this article will be made available by the authors, without undue reservation.

## Ethics statement

The studies involving human participants were reviewed and approved by Regional Ethics Committee - CER Liguria: 633/2020–DB id 11064. The patients/participants provided their written informed consent to participate in this study.

## Author contributions

MoB, AE, MaB, and MA contributed to conception and design of the study. MoB, AE, AT, FD, and BS recruited the subjects and performed research. AB developed the analysis software. MM performed the statistical analysis. AT wrote the first draft of the manuscript. MM and GS wrote sections of the manuscript. All authors contributed to the article and approved the submitted version.
