# Supplementary material for: “Surrounded, detached”: the relationship between defensive peripersonal space and personality
Source: Front Psychiatry. 2023 Oct 13;14:1244364. doi: 10.3389/fpsyt.2023.1244364 (PMC10603239; doi:10.3389/fpsyt.2023.1244364)
Supplement: Supplementary file 1 [file Data_Sheet_1.pdf]

Analysis of HBR responses

We performed three repeated-measures ANOVA to investigate area, onset latency and duration of the responses, all with factors ANGLE (three levels: FAR, MIDDLE and NEAR) and RECORDING SIDE (two levels: ipsilateral eye and contralateral eye).

Results for data concerning the Area Under the Curve (AUC) showed a main affect for the ANGLE ($F_{(2,108)} = 12.94$, $p = 0.0001$) as well as SIDE ($F_{(1,54)} = 11.85$, $p = 0.0011$) and no significant interaction ($p = 0.7887$).

Newmann-Keuls post-hoc showed a significant increase of AUC values in NEAR with respect to FAR ($p = 0.0001$) and MIDDLE ($p = 0.0002$). No significant difference between FAR and MIDDLE ($p = 0.4833$). Also, significant grater AUC values were found for the ipsilateral side than contralateral eye ($p = 0.0012$).

Results for data concerning the onset latency showed a main affect for the ANGLE ($F_{(2,108)} = 16.75$, $p < 0.0001$) as well as SIDE ($F_{(1,54)} = 249.79$, $p < 0.0001$) and no significant interaction ($p = 0.6201$).

Newmann-Keuls post-hoc showed a significant decrease of onset latencies in NEAR with respect to FAR ($p < 0.0001$) and MIDDLE ($p < 0.0001$). No significant difference between FAR and MIDDLE ($p = 0.2214$). Also, HBR responses were significntly slower in the controlateral side with respect to ipsilateral ($p < 0.0002$).

Results for data concerning the duration of the HBR showed a main affect for the ANGLE ($F_{(2,108)} = 21.63$, $p < 0.0001$) as well as SIDE ($F_{(1,54)} = 30.276$, $p < 0.0001$) and no significant interaction ($p = 0.7112$).

Newmann-Keuls post-hoc showed a significant increase of duration values in NEAR with respect to FAR ($p < 0.0002$) and MIDDLE ($p < 0.0001$). No significant difference between FAR and MIDDLE ($p = 0.1732$). Also, significant longer responses were found for the ipsilateral side with respect to contralateral eye ($p = 0.0012$).

Table S1. Comparison of Bayesian multilevel models of HBR data

Model	Intercept only	Linear	Polynomial	**Piecewise linear**	Exponential
	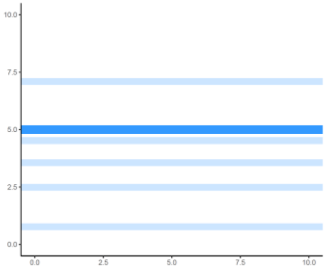	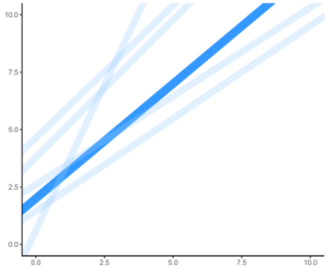	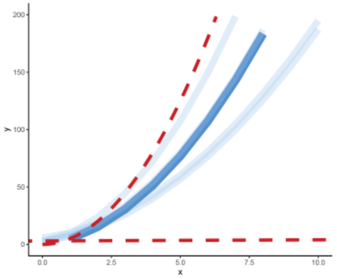	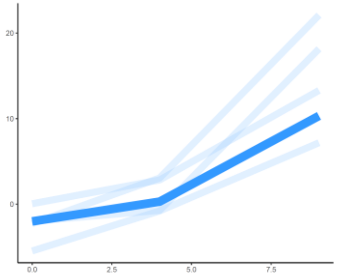	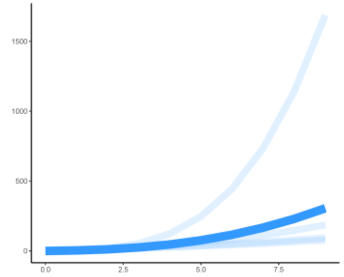
Brms formula	Alfa ~ 1 + (1 | id)	Alfa ~ 1 + angle + (1 + angle | id)	Alfa ~ 1 + angle + I(angle ^2) + (1 + angle + I(angle ^2) | id)	**Alfa ~ 1 + time + time_knot + (1 + time + time_knot | id))**	Alfa ~ b1 + b2 * angle ^ (b3),<br>b1 ~ 1 + (1|id), b2 ~ 1 + (1|id), b3 ~ 1 + (1|id)
R^2	74.7%	86.7%	88.7%	**89.1%**	92.4%
Elpd loo ± SE	-471.8 ± 15.6	-445.2 ± 12.6	-438.9 ± 13.6	**-437.7 ± 13.9**	-514.3 ± 17.8
Looic ± SE	944 ± 31.3	890.3 ± 25.3	877.8 ± 27.1	**875.3 ± 27.7**	1028.6 ± 35.5
Elpd_diff	-34.1 ± 8.0	-7.5 ± 5.0	-1.2 ± 1.1	**0.0 ± 0**	-3.7 * 10^{158} ± Inf

Elpd_loo: estimate of the Expected Log Pointwise Predictive Density (higher values indicate better fit). LOOIC: Leave-One-Out Information Criterion (LOOIC), lower values indicate better fit

Table S2. Parameters of the piecewise multilevel model of HBR data

	Estimate	Est.Error	l-95% CI	u-95% CI	Rhat	Bulk_ESS	Tail_ESS
Population level effects							
**Intercept**	2.727	0.067	2.598	2.860	1.004	1808.802	3974.479
**FtoM slope**	0.019	0.034	-0.047	0.086	1.000	10524.634	11783.780
**MtoN slope**	0.182	0.057	0.070	0.294	1.000	13739.787	11990.957
Group level effects							
**sd(Intercept)**	0.477	0.052	0.388	0.588	1.001	3331.332	6388.893
**sd(FtoM slope)**	0.090	0.042	0.016	0.189	1.005	1130.040	659.905
**sd(MtoN slope)**	0.110	0.081	0.005	0.311	1.008	781.356	594.897
**cor(Intercept,FtoM slope)**	0.489	0.284	-0.133	0.933	1.001	4985.309	7259.073
**cor(Intercept,MtoN slope)**	-0.134	0.423	-0.863	0.763	1.001	11288.095	8790.121
**cor(FtoM slope,MtoN slope)**	-0.253	0.469	-0.902	0.777	1.002	3443.855	9957.483
Family Specific Parameters							
**sigma**	0.156	0.019	0.109	0.190	1.007	896.131	435.206

Population level effects. The coefficient for FtoM slope represents the change in the expected value (HBR AUC) when transitioning from the far position to the middle position. This change can be thought of as the "slope" of the HBR AUC from the far position to the middle position. The coefficient for FtoM represents the change in the slope of the HBR AUC from the middle position to the near position.

Group level effects. sd(Intercept): Standard deviation of the intercepts across the different subjects (ID levels). sd(FtoM slope): Standard deviation of the random effect for the linear FtoM slope predictor. sd(MtoN slope): Standard deviation of the random effect for the piecewise FtoM slope_knot predictor at the middle position. cor(Intercept, FtoM slope): Correlation between the intercepts and the random effects for the linear FtoM slope predictor. cor(Intercept, MtoN slope): Correlation between the intercepts and the random effects for the piecewise FtoM slope_knot predictor at the middle position. cor(FtoM slope, MtoN slope): Correlation between the random effects for the linear FtoM slope predictor and the piecewise FtoM slope_knot predictor at the middle position.

Family Specific Parameters. sigma: Estimated dispersion parameter of the log-normal distribution, representing the variability of individual observations around the predicted mean response.

Figure S1. Parameters of the piecewise linear model

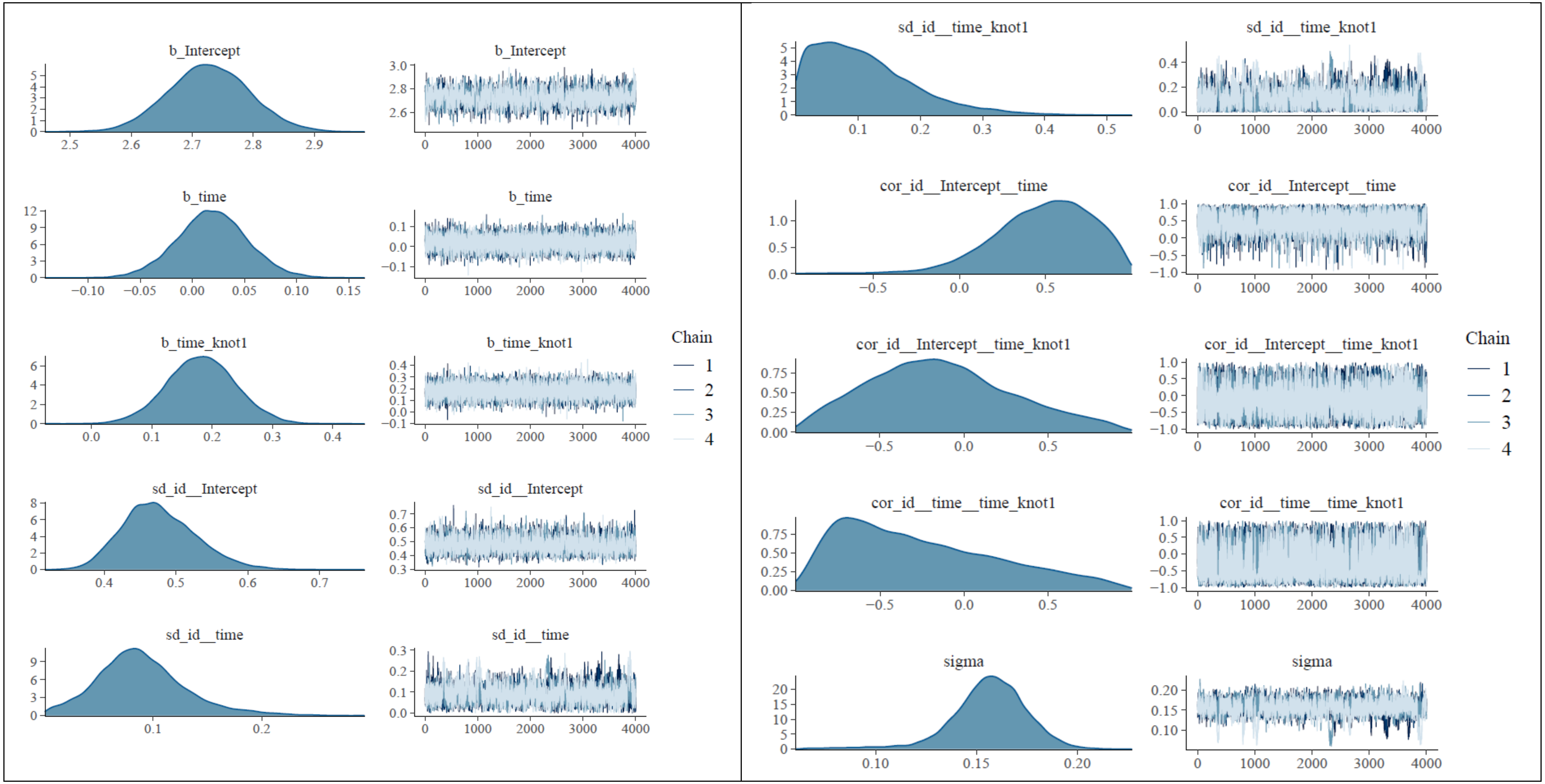

Figure S2. Posterior predictive checks of the Piecewise linear model

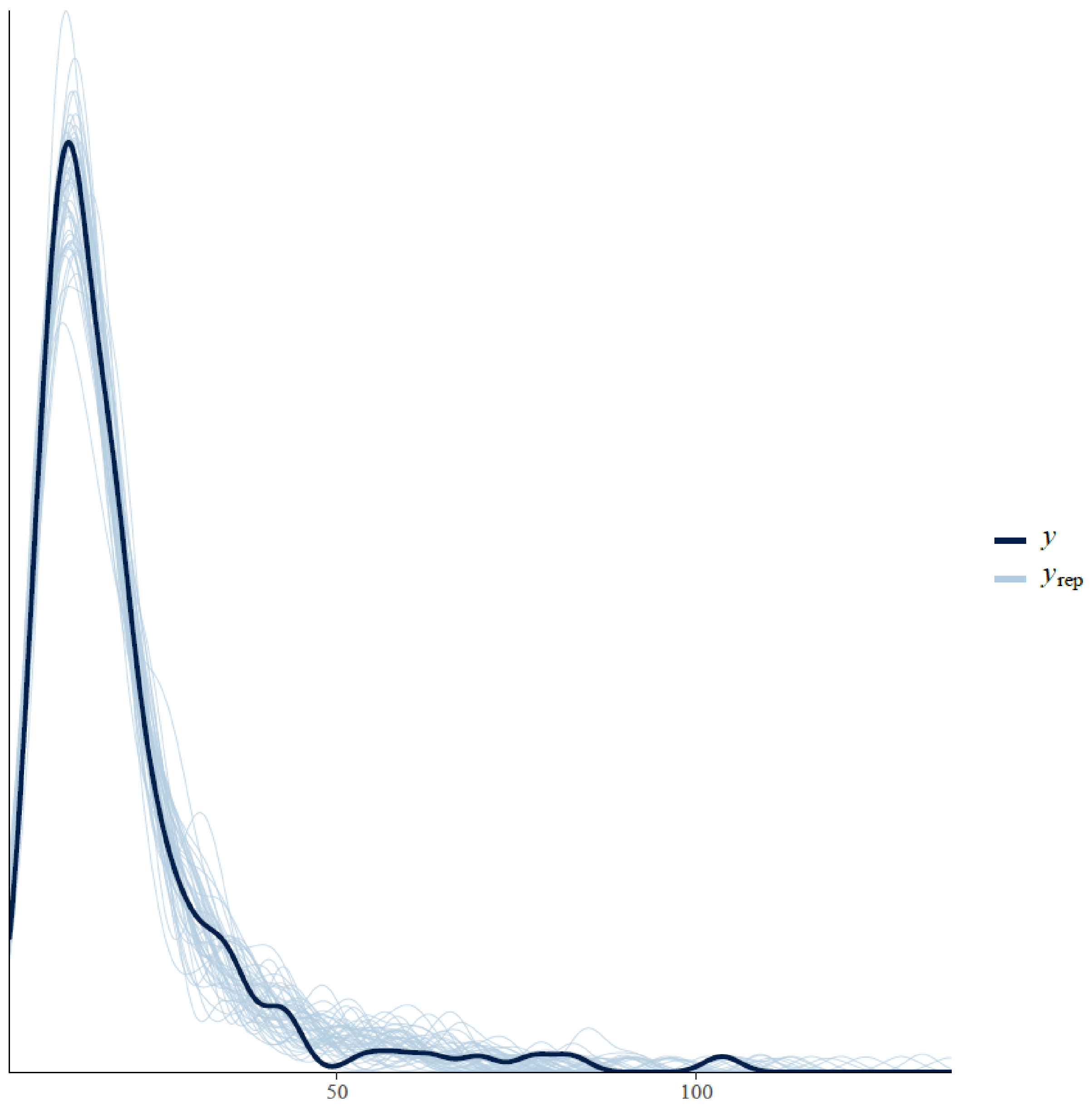

Table S3. Bayesian Hypothesis Testing for the absence (H0), presence of positive (H1) or negative edges in the network

	Relation	Post.mean	Post.sd	Pr.H0	Pr.H1	Pr.H2
**1**	**intercept--FtoM**	**0.914**	**0.171**	**0**	**1**	**0**
**2**	**FtoM--MtoN**	**0.525**	**0.175**	**0**	**0.999**	**0.001**
**3**	**Disinhib--Psychoticism**	**0.48**	**0.174**	**0**	**0.997**	**0.003**
**4**	**Antagon--Psychoticism**	**0.346**	**0.174**	**0.011**	**0.966**	**0.023**
**5**	**Detach--Antagon**	**0.324**	**0.169**	**0.016**	**0.957**	**0.027**
**6**	**Neg_Aff--Disinhib**	**0.313**	**0.168**	**0.02**	**0.949**	**0.031**
**7**	**Detach--Disinhib**	**0.265**	**0.174**	**0.057**	**0.883**	**0.061**
**8**	**Detach--Psychoticism**	**0.246**	**0.172**	**0.075**	**0.854**	**0.071**
**9**	**MtoN--Detach**	**0.242**	**0.174**	**0.081**	**0.852**	**0.075**
**10**	Neg_Aff--Antagon	0.197	0.166	0.14	0.759	0.101
**11**	FtoM--Disinhib	0.197	0.176	0.145	0.744	0.112
**12**	Neg_Aff--Detach	0.123	0.169	0.275	0.555	0.17
**13**	intercept--Detach	0.117	0.174	0.279	0.54	0.181
**14**	FtoM--Psychoticism	0	0.176	0.372	0.313	0.314
**15**	intercept--Antagon	-0.006	0.175	0.376	0.303	0.321
**16**	intercept--Psychoticism	-0.009	0.175	0.375	0.3	0.325
**17**	intercept--Neg_Aff	-0.015	0.17	0.388	0.284	0.328
**18**	FtoM--Antagon	-0.027	0.176	0.366	0.278	0.356
**19**	MtoN--Neg_Aff	-0.026	0.173	0.377	0.275	0.348
**20**	MtoN--Psychoticism	-0.028	0.173	0.374	0.272	0.354
**21**	Neg_Aff--Psychoticism	-0.027	0.172	0.379	0.271	0.35
**22**	FtoM--Neg_Aff	-0.051	0.17	0.367	0.242	0.391
**23**	FtoM--Detach	-0.064	0.175	0.345	0.233	0.422
**24**	MtoN--Disinhib	-0.118	0.176	0.275	0.183	0.542
**25**	MtoN--Antagon	-0.126	0.176	0.262	0.174	0.564
**26**	Antagon--Disinhib	-0.137	0.171	0.248	0.159	0.593
**27**	intercept--Disinhib	-0.178	0.175	0.176	0.128	0.696
**28**	intercept--MtoN	-0.651	0.172	0	0	1

The Bayes factor based methods allows for determining the conditional independence structure (evidence for the null hypothesis). The network depicts those associations where the posterior probability of the edge value being higher than 0 was 85% or higher.
